# On Growth and Morphology of TiO_2_ Nanotubes on Ti6Al4V by Anodic Oxidation in Ethylene Glycol Electrolyte: Influence of Microstructure and Anodization Parameters

**DOI:** 10.3390/ma14102540

**Published:** 2021-05-13

**Authors:** Bruno Ribeiro, Ruben Offoiach, Ehsan Rahimi, Elisa Salatin, Maria Lekka, Lorenzo Fedrizzi

**Affiliations:** 1 Lima Corporate, Via Nazionale 52, 33038 San Daniele del Friuli, Italy; bruno.ribeiro@limacorporate.com (B.R.); Elisa.Salatin@limacorporate.com (E.S.); 2 Polytechnic Department of Engineering and Architecture, University of Udine, Via Cotonificio 108, 33100 Udine, Italy; ruben.offoiach@uniud.it (R.O.); rahimi.ehsan@spes.uniud.it (E.R.); lorenzo.fedrizzi@uniud.it (L.F.); 3 CIDETEC, Basque Research and Technology Alliance (BRTA), Po. Miramón 196, 20014 Donostia-San Sebastián, Spain

**Keywords:** Ti6Al4V alloy, TiO_2_ nanotubes, anodization mechanism, morphology, ethylene glycol electrolyte

## Abstract

Different studies demonstrated the possibility to produce TiO_2_ nanotubes (TNTs) on Ti6Al4V alloy by electrochemical anodization. However, the anodizing behavior of α and β-phases in organic electrolytes is not yet clarified. This study reports on the anodizing behavior of the two phases in an ethylene glycol electrolyte using different applied potentials and anodizing times. Atomic force and scanning electron microscopies were used to highlight the anodic oxides differences in morphology. It was demonstrated that the initial compact oxide grew faster over the β-phase as the higher Al content of the α-phase caused its re-passivation, and the higher solubility of the V-rich oxide led to earlier pores formation over the β-phase. The trend was inverted once the pores formed over the compact oxide of the α-phase. The growth rate of the α-phase TNTs was higher than that of the β-phase ones, leading to the formation of long and well defined nanotubes with thin walls and a honeycomb tubular structure, while the ones grown over the β-phase were individual, shorter, and with thicker walls.

## 1. Introduction

The electrochemical anodization for the production of TiO_2_ nanotubes (TNTs) attracted a special interest in the last decades due to their outstandingly unique features and because it is a low cost method which allows direct control of nanotube shape and size with a good reproducibility [1]. Moreover, it has the advantage to produce vertically oriented nanotubes arrays directly on the surface of Ti alloys [2]. The interesting features of TNTs, ascribing to their crystal structure (anatase-rutile), large surface area (high aspect ratio), optical and electrical properties, photocatalytic activity, and bioactivity, make them a valid candidate for a large variety of applications such as electrochromic devices, dye-sensitized solar cell, Li-ion battery anodes, biosensors, osteointegration promoters, and antibacterial agents reservoirs for prosthetic implants [1,2,3,4,5,6,7].

After the work of Zwilling et al. in 1999 [8] that established the presence of F^−^ ions in the electrolyte as a key factor to grow vertically oriented nanotubes arrays directly on the surface of Ti alloys, a large amount of research work was done by different groups in order to optimize the TNTs synthesis process. The influence of the electrochemical parameters (current or potential control, ramp of applied voltage, applied potential difference, time, temperature, stirring, double step anodization approaches, etc.) and the use of different electrolyte compositions [1,9] as well as their aging [9,10] were explored. Most of the research works were largely carried out on pure titanium, establishing different approaches and procedures which allow a precise control of the morphology of the TNTs and also proposing gradually more accurate models to explain the growth mechanism and to better specify the role exerted by the F^−^ ions [1,9]. F^−^ ions and water are key ingredients for the TNTs’ successful fabrication. The F^−^ ions form water-soluble fluoro-complexes (TiF_6_^−^) both by complexing the Ti^4+^ ions and by chemically attacking the formed TiO_2_. This directly influences the anodization current evolution under a constant potential. Several studies showed that there is a strict correlation between the current–time transient (i–t curves) and the different stages of TNTs growth by performing potentiostatic anodization of pure Ti under specific conditions. In general, it is usual to distinguish three different stages: *Stage I*—formation of a compact oxide layer (sharp decrease of the current), *Stage II*—pores nucleation exerted by F^−^ ions (moderate increase of the current in short time), *Stage III*—growth of TNTs under a dynamic equilibrium between the oxide formation and the metal/oxide dissolution (quasi-steady-state values of the current) [1,5,11]. This dynamic equilibrium that allows producing “self-organized structures”, nevertheless, largely depends on the chemical composition of the electrolyte and the electrochemical parameters [1,5,12].

Nowadays, it is common to distinguish the different anodizing electrolytes in two big categories: water based (or aqueous) electrolytes, first to be studied, and organic based electrolytes [9]. A first generation of TNTs, based on the initial work of Zwilling, was grown in aqueous HF electrolytes, where the chemical dissolution of both the Ti substrate and TiO_2_ was quite significant. The produced nanotubular layers were characterized by having only a few microns in length, a low degree of organization, and considerable side wall inhomogeneity due to thickness variations or ripples, which were the result of oxygen evolution reactions. Different attempts were done later to adjust the composition in order to reduce the dissolution effect by using both different F^−^ ions sources and different supporting reagents. To this aim, a second generation of TNTs was achieved optimizing the F^−^ ions content, varying the electrolyte pH, and using different cations. It was demonstrated that longer and more self-organized TNTs can be obtained by reducing the electrolyte acidity, using, for example, fluoride salts instead of HF, and that some cations, such as NH_4_^+^, can positively influence the growth mechanism [13,14,15,16].

Organic electrolytes containing small amounts of fluoride ions (0.1–0.5 wt.%) and water (0.1–5 wt.%) attracted significant interest by virtue of their capability to produce longer and highly ordered TNTs arrays (third generation of TNTs), influencing markedly the process kinetics owing to lower ionic mobility. Different organic media were investigated such as glycerol, formamide, ethylene glycol, dimethyl sulfoxide (DMSO), and N-methylformamide [9,12,17,18,19,20,21]. The water content is a crucial factor in organic electrolytes since it affects markedly both the growth rate, being practically the sole supplier of oxygen ions for the oxide formation, and the dissolution rate, acting as solvent for water-soluble metal fluorides. Moreover, in organic media, the aggressiveness of F^−^ ions is mitigated, directly influencing the dissolution rate. All these factors under optimized conditions allow to extend markedly the anodization time (up to tens of hours) and therefore to increase the TNTs length up to several hundreds of microns. Due to the limited dissolution in this type of electrolyte, a “compact porous layer” can remain on the top of the TNTs for longer anodization times [9]. This “compact porous layer” is what remains of the initial compact oxide layer, formed in the early stages of the anodization, and, during the growing stage, prevents the fast dissolution of the TNTs that are growing under it. When this layer is completely dissolved, after longer anodization times, the action of F^−^ ions can cause a marked thinning of the tube’s top walls, causing a collapse of the nanotubular structure and leading to the formation of a morphology named “nanograss”, which has a deleterious effect on the TNTs properties [1]. A detailed description of these morphological transitions and an accurate study of the growth kinetics which govern the above mentioned different stages (*Stage 1*—nanotube growth under a gradually dissolving “compact porous layer”; *Stage 2*—short period in which the “compact porous layer” is complete dissolved leaving the tubes top open; *Stage 3*—advancement of the tubes top dissolution and progressive “nanograss” formation) was recently given by Seçkin and Urgen, studying the anodization process of pure Ti foils in an ethylene glycol based electrolyte containing NH_4_F (0.3–0.6 wt%) and H_2_O (1 vol%) [21].

For organic electrolytes, the majority of the studies on the growth mechanisms of TNTs are in regard to pure Ti and, hence, not much information is available regarding the TNTs growing over more complex systems such as binary, ternary, or Ti alloys with more than three compounds [1]. The microstructure and the chemical composition of Ti alloys affect markedly the TNTs growth, since the different phases can undergo different etching and anodizing rates [1,22]. Moreover, the formation of well-organized nanotubular arrays free of inhomogeneity over a large surface area can be achieved apparently only on pure Ti or, more in general, on single phase titanium alloys [22].

Among the vast assortment of titanium alloys, the ternary Ti6Al4V alloy is one of the most technologically relevant, mainly in the biomedical field, and different studies demonstrated the possibility to produce TNTs on it using both water based [8,23,24,25,26] and organic based electrolytes [27,28,29,30,31]. In water based electrolytes, starting from Zwilling [8], different studies [23,24,25,26] highlighted that the biphasic microstructure of the underlying alloy (α-phase enriched in Al and β-phase enriched in V) affected markedly the size and the distribution of pores in the anodic oxide layer. Most of the works reported that the α-phase region presented a self-organized nanotubes array, while the β-phase region presented an irregular nanoporous structure [8] or underwent complete dissolution [22]. In particular, Macak et al. [23], by performing potentiostatic anodization at the same conditions on Ti6Al4V and Ti6Al7Nb, pointed out that TNTs could grow only on β grains enriched in Nb and not on β grains enriched in V, attributing this behavior to the high solubility of V-rich anodic oxide and, hence, its early passivity breakdown. The use of organic electrolytes for the anodization of Ti6Al4V instead was reported in few works, and most of them were related to ethylene glycol based electrolytes containing NH_4_F and water [27,28,29,30,31]. Among them, only the work of Jordanovová et al. [31] clearly showed the differences on the morphology of the anodic oxides grown over α and β-phases.

This study aimed to clarify the anodization behavior of α and β-phases of a Ti6Al4V alloy. To this purpose, potentiostatic anodizations were performed in an ethylene glycol electrolyte containing 0.5 wt.% NH_4_F and 2.5% V H_2_O using different applied potentials and anodization times. The effect of these parameters on the morphology of the TNTs over both phases was investigated. Moreover, in order to better understand the TNTs growth mechanism on α and β-phases, short-time anodizations, from seconds to minutes, were carried out, and the anodic oxide evolution was investigated by field emission scanning electron microscopy (FESEM) and atomic force microscopy (AFM).

## 2. Materials and Methods

### 2.1. Ti6Al4V Specimen Preparation

Disks with a thickness of 4 mm and a diameter of 30 mm were cut from a rod of Ti6Al4V ASTM F1472 alloy (ATI Europe Distribution-Allegheny Technologies GmbH, Remscheid, Germany), the chemical composition (wt.%) of which was 6.25 Al, 0.065 C, 0.23 Fe, 0.003 H, 0.01 N, 0.185 O, 4.45 V, 0.001 Y, Ti (balance); these were used as substrates for all experiments. The disks were ground using SiC papers (grades 800 up to 4000) and then mechanically polished using a silica suspension (OP-S NonDry silica suspension 0.25, STRUERS with 25% H_2_O_2_). After polishing, all samples were ultrasonically cleaned for 5 min in ethanol, acetone, and DI water in sequence and finally dried by air blowing. For the metallographic investigations, polished samples were chemically etched for 15 s using Kroll’s etchant (2% *v*/*v* HNO_3_ (68%), 1% *v*/*v* HF (48%).

### 2.2. Electrolytic Anodization Process

The electrolyte used for the anodization was an ethylene glycol solution containing 0.5% wt. NH_4_F and 2.5% V DI H_2_O, which was kept at room temperature. All electrolyte batches were prepared using high purity reagents (ethylene glycol anhydrous 99.8%, ammonium fluoride, 98.0%, SIGMA-ALDRICH, St. Louis, MO, USA) and DI water. The electrolytes were aged by anodizing on sacrificial specimens for 5 h at 60 V prior to the anodization of the analyzed specimens. The time and the applied potential for the aging were optimized based on preliminary tests to check the stability of the electrolyte and the reproducibility of the anodizing process. The anodic oxidation was carried out in a two-electrode setup using a DC power supply. The Ti6Al4V disks were used as the anode, while a platinum plate was used as the cathode. A polycarbonate cell was fabricated to this aim in order to ensure a 2 cm anode–cathode distance and a circular area of both anode and cathode exposition to the electrolyte with a diameter of 2 cm. Silicone O-rings were used for this purpose and to prevent leakage.

Nanotubes layers were produced varying the anodization time (30, 60, and 120 min) and the voltage (20, 40, 60 80, and 100 V) in order to study the effect of these parameters on the layer morphology. To study the early steps of the anodization process and clarify the kinetics of growth on the two different phases of Ti6A4V, additional anodization experiments were conducted at 60 V for times of 25 s, 40 s, 120 s, 300 s, 900 s, and 1500 s. After anodization, the samples were cleaned by utrasonication in acetone for 3 min and air dried.

### 2.3. Surface Analysis and Morphology Characterization

The microstructure of the Ti6Al4V alloy and the morphology of the anodized specimens were assessed by field-emission electron microscopy (FESEM) (JEOL model JSM-7610F Plus (JEOL Ltd. Tokyo, Japan) equipped with an Oxford X-MAX20 energy dispersive X-ray spectrometer (EDXS) (OXFORD Instruments, Abingdon, UK). Micrographs were captured in the middle region of the anodized area using a secondary electrons in-lens detector. In order to characterize the nanotube layer in cross section, the anodized samples were cut, hot-embedded in epoxy resin, and polished to mirror-like surface.

AFM topographic and Volta potential maps were obtained using a Digital Instruments Nanoscope IIIa Multimode (Bruker Corporation, Billerica, MA, USA) with an n-type doped silicon pyramid single-crystal tip coated with PtIr5 (SCM-Pit probe). The surface maps were captured in dual-scan mode. In the first scan, topography data were obtained in tapping mode, and in the second scan, the surface potential was determined by lifting the tip 100 nm. The measurements were performed in air atmosphere at room temperature and an approximate relative humidity of 28% at a scan frequency rate of 0.2 Hz with pixel resolution of 512 × 512 and zero-bias voltage.

## 3. Results

### 3.1. Microstructural Analysis of the Base Alloy

**Figure 1:**
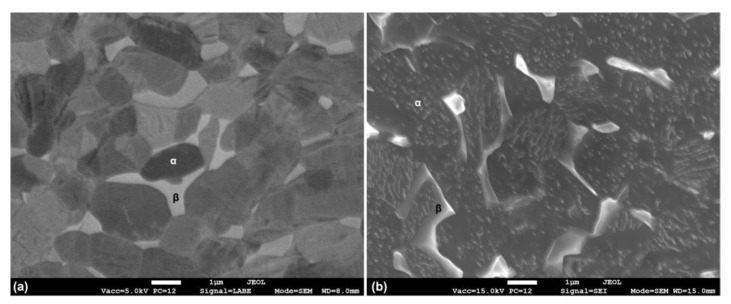
FESEM micrographs of the bare Ti6Al4V sample: (**a**) BSE micrograph, (**b**) SE micrograph after metallographic etching (Kroll’s solution 15 s).

Figure 1 shows FESEM micrographs of the Ti6Al4V samples polished in backscattered mode (Figure 1a) and after metallographic etching in Kroll’s solution for 15 s (Figure 1b). The Ti6Al4V samples presented a polycrystalline structure with grains of different sizes. EDXS analyses on different areas (Table 1) identified, as the α-phase, the darker areas of Figure 1a (richer in Al), while the elongated brighter grains, which presented a relatively higher concentration of V, were identified as β-phase grains. Following the metallographic etching, the two phases could be more clearly distinguished (Figure 1b) as the higher amount of V in the β-phase decreased the reactivity of the alloy to the etchant.

**Figure 2:**
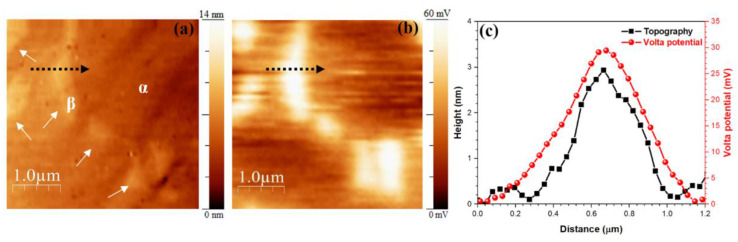
(**a**) AFM topography and (**b**) SKPFM Volta potential maps of polished Ti6Al4V alloy, (**c**) line profiles along the black dashed arrow of (**a**,**b**).

AFM topographic and SKPFM Volta potential maps of a polished Ti6Al4V sample together with the line profiles are reported in Figure 2. β-phase grains (see white arrows in Figure 2a) appeared slightly higher at the topographic map (~3 nm), possibly due to the difference in hardness having an effect on the polishing procedure. The different chemical composition between α-phase and β-phase was reflected in a difference of approximately 30 mV in the Volta potential (Figure 2b,c) with the β-phase exhibiting the higher values, in accordance with what was previously reported by other authors [16,32]. Indeed, V had a higher WFE, 4.3 eV, in comparison to Al, which had a WFE of 4.26 eV in the polycrystalline condition [33]. The β-phase, which had a higher V content, had a higher relative surface potential and acted as a nobler phase than the α-phase, which had a higher Al content.

### 3.2. Morphology of TiO_2_ Nanotubes

**Figure 3:**
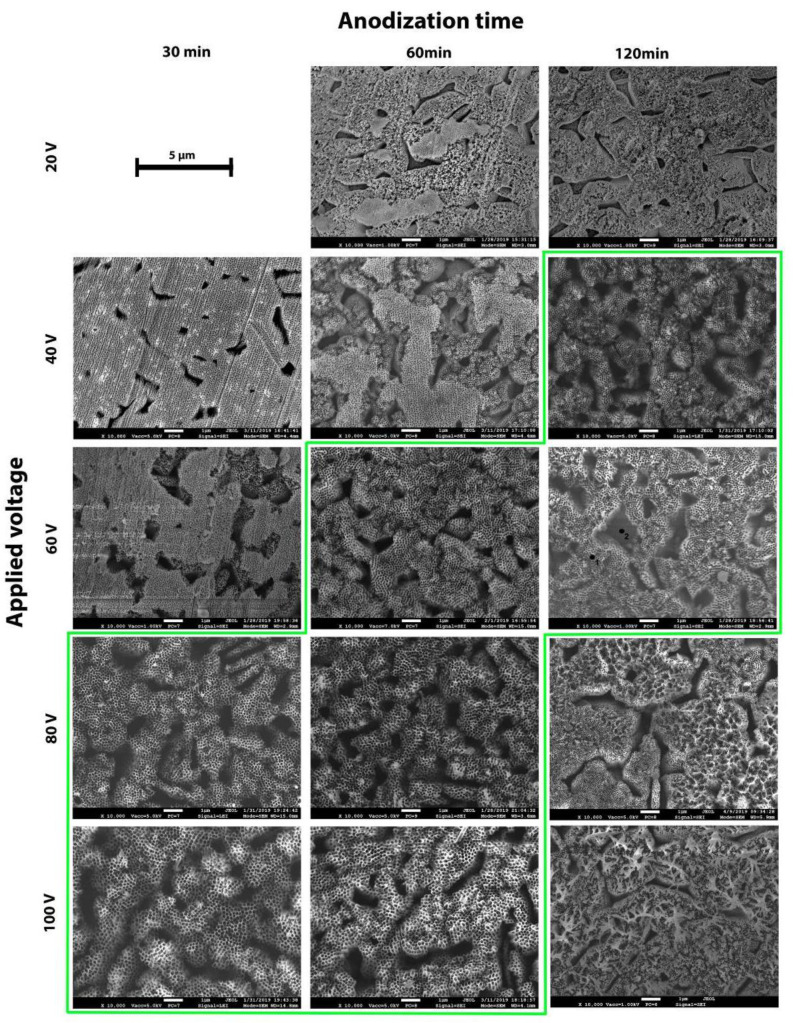
FESEM micrographs depicting a top-view of the anodic oxide layer produced through electrochemical anodization at different applied potentials and anodization times. The green frame shows the conditions with open-mouth nanotubes.

Figure 3 shows FESEM micrographs depicting a top view of Ti6Al4V samples anodized using different applied potentials and times.

From the low magnification micrographs, it was clear that the anodic oxide was not uniform over the whole surface and presented large cavities, the shape, the size, and the distribution of which closely resembled those of the β-phase grains (Figure 1b). EDXS analyses were performed inside and outside these cavities, for example, on the areas marked on the micrograph referring to the specimen anodized at 60 V for 120 min in Figure 3 as 1 (upper surface) and 2 (cavity). The results are reported in Table 2. Due to differences in height of the analyzed areas and the narrow shape of the cavities, the results were considered qualitative. In order to minimize the effect of the shape, EDXS measurements were performed on different sites, and the values presented in Table 2 are the mean values of 15 distinct measurements.

The higher V content on the cavities and the higher Al content on the upper surface confirmed the hypothesis that the cavities corresponded to anodic oxide grown over the β-phase grains.

Focusing on the morphology of the anodic layer over the α-phase, we could clearly observe that the obtained oxide was porous and non-uniform. The observed differences in morphology by changing voltage and time were clearly attributed to the advance of the anodization process. The increase in voltage and the increase in time usually led to increased dissolution. The applied potential difference determined the strength of the applied electric field, directly influencing the ion migration rate and therefore the dissolution rate. On the other hand, the longer the time was, the longer the dissolution was [1]. In accordance with the work of Seçkin and Ürgen [21], it became clear that these differences in morphology represented different stages of anodization.

The anodization at 20 V for 30 min (not reported) did not produce any porous layer. The anodic oxide produced at 40 V for 30 min was a porous compact layer, which was clearly in *Stage 1*. On the other hand, the samples anodized at 20 V for 60 and 120 min, at 40 V for 60 min, and at 60 V for 30 min presented only remnants of this porous compact layer, and it was possible to observe the presence of defined nanotubular structures in the areas where it dissolved. These results clearly demonstrated that nanotubular structures existed below this compact porous oxide, which is in accordance with previous findings of other researchers [7,34] and further supports the model of morphological development proposed by Seçkin and Ürgen [21]. Well-defined nanotubes were obtained on the samples highlighted by the green line in Figure 3, namely, 40 V for 120 min, 60 V for 60 and 120 min, and 80 V and 100 V for 30 and 60 min. For each of these conditions, it was possible to see that the tube tops were fully open and well defined. Thus, these samples could be considered at the *Stage 2* of anodization according to Seçkin and Ürgen [21], when defined nanotubular structures are seen and before the dissolution of the tube tops with the marked effect of the formation of nanograss. Indeed, a further increase of time at high applied voltage (80 or 100 V) led to the formation of nanograss due to the extensive thinning of the nanotubes walls (*Stage 3* according to Seçkin and Ürgen [21]).

As mentioned before, one particular aspect of the produced anodized layers over the Ti6Al4V alloy was the non-uniform growth of the nanotubes over α and β phases. This lack of uniformity was previously reported by several authors, confirming that, over the α-phase, a well-defined nanotubular structure is generally grown, while, over the β-phase, pits are observed with an oxide not quite developed, which may or may not present a nanotubular structure, depending on the anodization process conditions [14,19,26,27,31].

**Figure 4:**
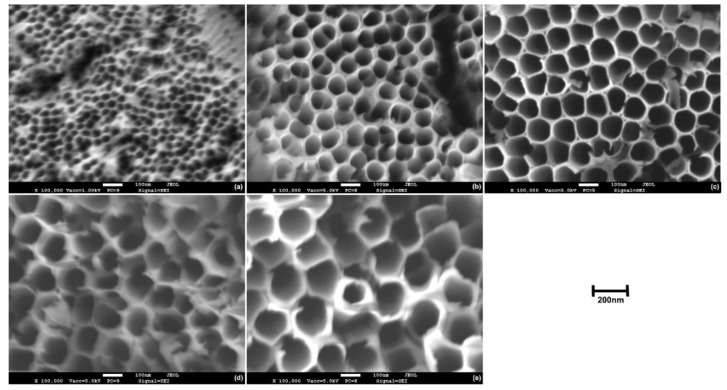
High magnification FESEM micrographs depicting a top-view of the anodic oxide layer produced over the α-phase through electrochemical anodization for 60 min at (**a**) 20 V, (**b**) 40 V, (**c**) 60 V, (**d**) 80 V, and (**e**) 100 V.

**Figure 5:**
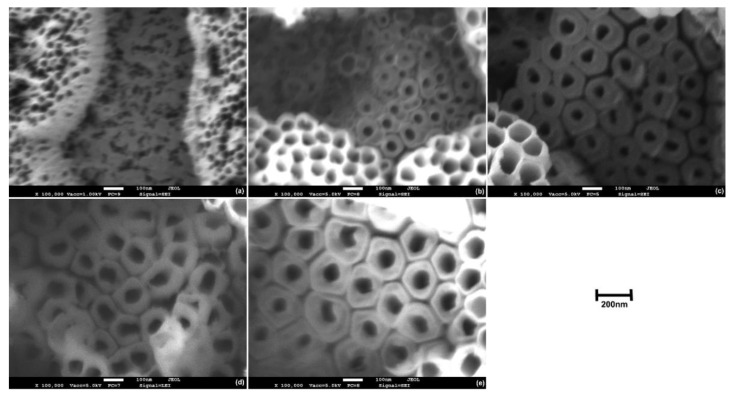
High magnification FESEM micrographs depicting a top-view of the anodic oxide layer produced over the β-phase through electrochemical anodization for 60 min at (**a**) 20 V, (**b**) 40 V, (**c**) 60 V, (**d**) 80 V, and (**e**) 100 V.

On the other hand, not all authors recognize the dual nature of morphology of the oxide produced over the Ti6Al4V alloy, which could be related to the different compositions of the electrolytes used in the anodization processes [31,35,36]. In Figure 4 and Figure 5, FESEM micrographs at high magnifications of the anodic oxides over α- and β-phases, respectively, are reported for specimens anodized for 60 min at different applied potentials. It was possible to observe that the produced oxide presented a dual morphology, in line with previous reports. The nanotubes grown over the α-phase presented a well-defined honeycomb-like tubular structure with thin walls and with inner diameters, which increased by increasing the applied voltage (Figure 4). At high applied potentials (80 V and 100 V), the tops of the TNTs walls appeared partially fractured due to the extensive thinning. The oxide grown over the β-phase at 20 V did not present an organized tubular structure (Figure 5a). Poorly organized TNTs were observed at 40 V (Figure 5b), and a further increase of the potential increased the TNTs’ organization degree (Figure 5c–e). The nanotubes obtained over the β-phase presented generally much thicker walls and smaller inner diameters with respect to those produced under the same conditions over the α-phase.

The early works by Zwilling et al. also reported on this phenomenon, attributing it to the higher electrochemical stability of the V rich grains, which resulted in lower growth rates of the anodic oxide [8,37]. This was later corroborated by other authors [38,39]. On the contrary, a recent work [31] reported that the growth rate over the β-phase was faster due to the quicker diffusivity into the BCC structure, but the controversial process of the TiO_2_ dissolution during the TNTs growth proceeded preferentially over the β-phase.

**Figure 6:**
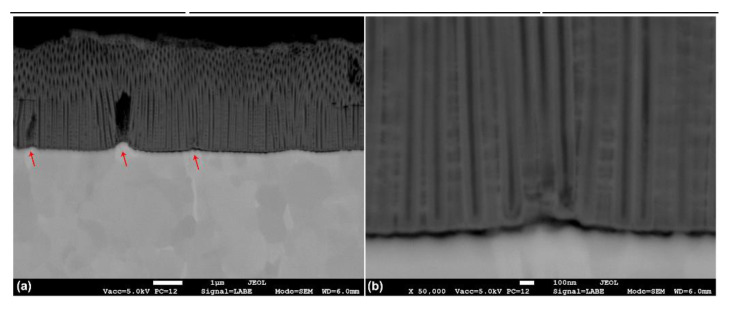
Cross section FESEM micrographs of samples anodized at 60 V for 60 min: (**a**) nanotubes over the two phases and (**b**) α-phase detail.

The FESEM micrograph in the cross section (Figure 6) clearly shows that β-phase grains (red arrows in Figure 6a) appeared higher than α-phase grains, indicating the lower dissolution rate of the V-rich phase. The nanotubes over the β-phase grains were much shorter and almost invisible in the cross section, while the nanotubes growing over the α-phase were well defined and presented smooth tube walls, as indicated on the FESEM micrograph at higher magnifications (Figure 6b). Thus, these results further support the notion that nanotube growth over the β-phase occurs at a lower growth rate.

**Figure 7:**
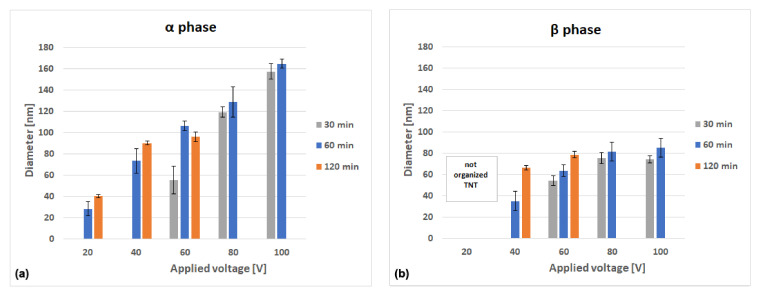
Average inner nanotubes diameter as a function of applied potential and anodization time for the conditions where open nanotubes were observed. (**a**) α-phase nanotubes and (**b**) β-phase nanotubes.

To better understand how the morphology changes with the variation of the applied potential difference and time, the inner nanotube diameters were measured for all the tested samples on which surface areas with completely open nanotubes were encountered, even at low extension. The results are reported in Figure 7.

Regarding the nanotubular layer grown over the α-phase (Figure 7a), the diameter increased in an almost linear way with the increase of the applied voltage, keeping constant the anodization time, in line with previous research works [1,5,13]. For an anodization time of 30 min, the inner diameter measured was about 55 nm for 60 V, reaching 160 nm for 100 V. Similarly, for 60 min of anodization time, the inner diameter measured was about 75 nm for 40 V, reaching 165 nm for 100 V. For 120 min, the same trend was observed. The increase of anodization time under a constant applied potential difference also led to an increase of the nanotubes diameter, even at a lower extension. The inner diameter of the nanotubes grown over the β-phase (Figure 7b) was much lower compared to that of the α-phase nanotubes and did not exceed 85 nm for all tested conditions. The effect of voltage, keeping constant the time, was similar to that observed on α-phase nanotubes until 60 V. By further increasing the applied voltage, no significant increase of the diameter was observed. The effect of time, keeping constant the voltage, was marked at low potential differences (40 V and 60 V); instead, it was almost irrelevant at higher voltages (80 V and 100 V), showing a quasi-steady state of the variation of the diameter.

Pore diameter, inter tube spacing, and self-ordering are strongly related to pore nucleation during the initial phase of the growth process. The applied potential difference directly determines the strength of the electrical field and, hence, the ion migration rate and the dissolution rate. As such, they largely influence pore nucleation during the initial phase of the growth process, which in turn determines pore diameter, inter tube spacing, and self-ordering of the tubular structures. Thus, the direct relationship between the inner diameter and the voltage is expected and was extensively reported for TNTs grown on different Ti substrates [18,36,40,41,42,43].

On the other hand, increased anodization time leads to a longer dissolution process. It was demonstrated that not all pores formed during nucleation generated an individual tube. Some pores stopped growing at some point during the process and were incorporated into larger growing pores, thus increasing the final diameter. While an intense dissolution may lead to an increase of pore diameter in the early stages of tubular development, once *Stage 2* of tubular development is reached (according to Seçkin and Ürgen [21]), over-dissolution of the tube tops occurs, which could explain the inversion of the effect of time at 120 min of anodization for the samples produced at 60 V on the tubes grown on the α-phase. Thus, there is a limit on the effect of time, depending on the nature of the electrolyte. In aqueous electrolytes, the maximum thickness is normally achieved within a few minutes, but additional time (for a total between 30 min and 2 h) is required to allow the anodic oxide to rearrange itself and increase the degree of self-ordering. In organic electrolytes, the process is much slower, and the dissolution phenomena are not as significant; hence, the effect of increasing time on the diameter is less evident [9].

### 3.3. Growth Mechanism

**Figure 8:**
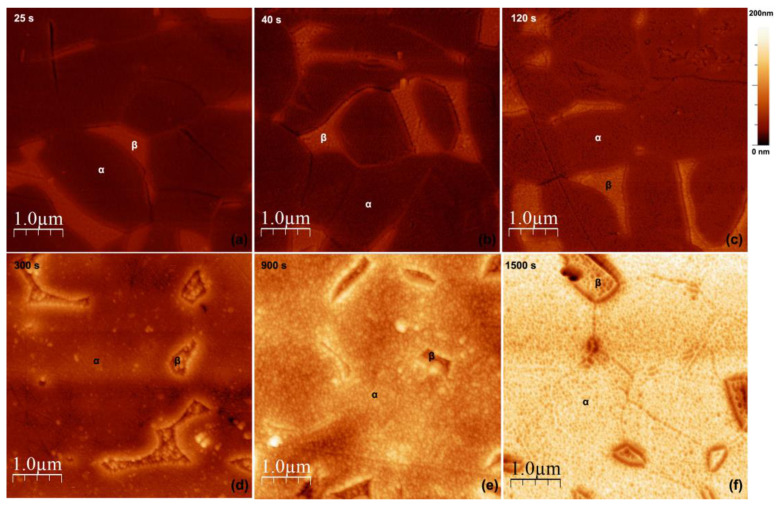
AFM topography maps for specimens anodized at 60 V at (**a**) 25 s, (**b**) 40 s, (**c**)120 s, (**d**) 300 s, (**e**) 900 s, and (**f**) 1500 s.

**Figure 9:**
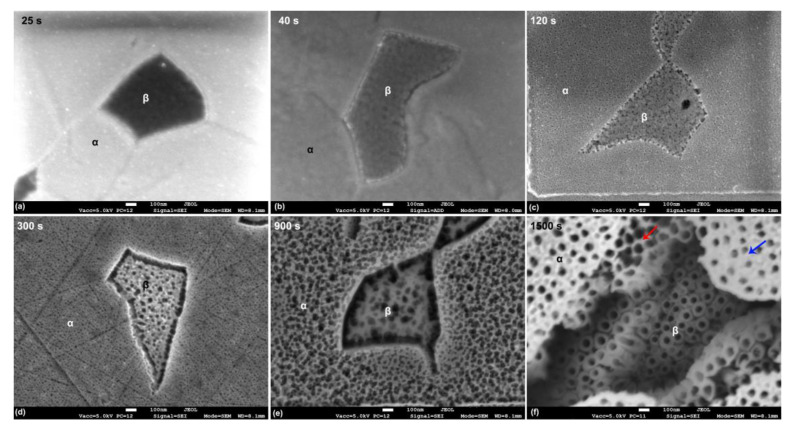
FESEM micrographs of samples anodized at 60 V at (**a**) 25 s, (**b**) 40 s, (**c**)120 s, (**d**) 300 s, (**e**) 900 s, and (**f**) 1500 s.

In order to better understand the growth mechanism of the TNTs over the different phases of Ti6Al4V, samples were anodized at 60 V (condition where high ordered nanotubes were observed over both phases) for different times, including very short ones, and were observed by FESEM and AFM. AFM topographic maps of the anodized specimens are reported in Figure 8, and FESEM micrographs of the same samples are shown in Figure 9.

AFM topography maps revealed that, after only 25 s of anodization (Figure 8a), the β-phase grains appeared higher than the α-phase grains, and the grain boundaries were more marked compared to the polished Ti6Al4V alloy surface (Figure 2a). This was an indication that an oxide as already formed over the surface and that it was thicker over the β-grains. The FESEM micrograph after the same anodization time confirmed the presence of a compact oxide over both phases (Figure 9a). By increasing the anodization time to 40 s, the height difference between α- and β-phase grains increased (Figure 8b), indicating a faster growth of the compact oxide over the β-phase during the early anodization stages. The FESEM micrograph (Figure 9b) confirmed the AFM results and revealed the initiation of the pores formation over the β-phase compact oxide. The α-phase oxide remained compact. After 120 s, the α-phase oxide started to grow more, and the β-phase oxide formation slowed down so that the difference in height became lower, with the β-phase grains still having the higher height (Figure 8c). The FESEM observations showed that pores started to appear on the oxide surface over the α-phase, while the ones over the β-phase became larger (Figure 9c). After 300 s, the height difference was inverted; the anodic oxide over the β-phase grains appeared lower than that over the α-phase (Figure 8d), indicating that the nanotubes were growing faster under the compact oxide layer on the α-phase than on the β-phase. The FESEM micrograph (Figure 9d) showed an increase on the pores diameter on the compact oxide layer over both phases, with the ones on the β-phase being still larger. The same trend was maintained after 900 s, with the anodic oxide growing faster over the α-phase, increasing the difference in height (Figure 8e). The dissolution process of the initially formed compact oxide continued over both phases, as demonstrated by the increase of the pores size (Figure 9e). After 1500 s, the nanotubes over the β-phase appeared completely open and much shorter than those over the α-phase (Figure 8f). On the α-phase, some areas with completely open nanotubes were visible (red arrow in Figure 9f), while on others, the initially formed compact oxide was still covering the nanotubes’ upper part (blue arrow, Figure 9f).

Considering the growth stages defined by the current–time transient model [1], it was clear from the AFM and the FESEM results that the growth of the anodic oxides proceeded with different speeds on the two alloy phases. The initial compact oxide (*Stage I*) grew faster over the β-phase, even if it presented a higher Volta potential, which meant that it should have been electrochemically more stable. This could be attributed to the chemical composition of the formed oxides over the two phases. According to previous studies [38], the higher content of Al in the α-phase in electrolytes containing high concentrations of F^-^ ions can lead to a re-passivation due to the formation of 4AlOF·TiOF_2_·H_2_O or AlF_3_, which is poorly soluble. On the other hand, the presence of a higher amount of V in the β-phase leads to the formation of a V-rich oxide, which grows faster and presents pores even after 40 s of anodization due to its higher solubility of V-oxides, resulting in unstable passivity and thus earlier breakdown (*Stage II*) [23,26,44].

The trend was inverted once the first pores were formed over the compact oxide of the α-phase. Highly ordered nanotubes grew over both phases, protected by the porous compact layers. The growth rate was faster over the α-phase than over the β-phase, as demonstrated by both AFM and FESEM results. The dissolution process on the bottom of the tubes due to the actions of the F^−^ ions was faster over the α-phase, while the initially formed compact oxide dissolved more slowly, protecting the integrity of the growing nanotubes. The initially formed compact oxide over the β-phase dissolved faster, but the integrity of the nanotubes was maintained, contrary to what was reported by other authors during anodization of Ti6Al4V in aqueous electrolytes [23,26]. The use of ethylene glycol electrolyte with a low amount of H_2_O slowed down the dissolution of the water soluble V-rich oxides and led to the formation of individual nanotubes, even after 1 h of anodization on the β-phase. The final result was the formation of highly ordered nanotubes over both phases, with different lengths, internal diameters, and wall thicknesses.

**Figure 10:**
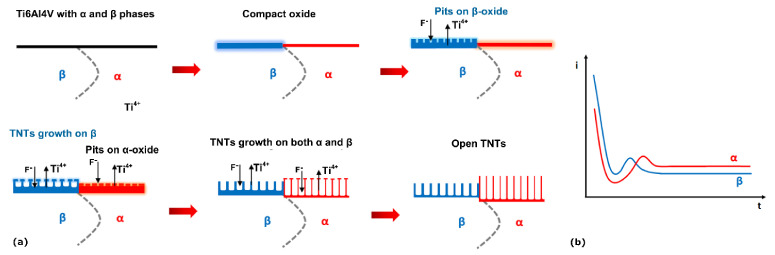
(**a**) Schematic representation of the different steps of growth of the anodic oxide layer in Ti6Al4V alloy. (**b**) Suggestion of the individual current density-time plot for each individual phase adapted from [1].

The steps of growth are illustrated schematically in Figure 10 together with the proposed current density-time transients considering the individual phases.

Briefly, under a constantly applied overall potential difference, the current density over the β-phase was initially higher than over the α-phase, leading to the formation of a thicker barrier oxide, followed by a steady decrease in current density on both phases (*Stage I*). At a certain point (before the first 40 s of anodization), there was a current peak corresponding to the pore nucleation on the β-phase oxide (*Stage II*). The α-phase oxide followed shortly after (before the first 120 s), and then both reached a steady current (*Stage III*) with a higher current density related to the nanotubes growing over the α-phase.

## 4. Conclusions

In this work, α- and β-phase anodization behaviors of a Ti6Al4V alloy in an ethylene glycol electrolyte were studied. Different applied potentials and anodization times were applied for this purpose, including very short ones, in order to understand the anodization mechanisms and kinetics of both phases.

The dual phase microstructure of the Ti6Al4V alloy led to the formation of a non-uniform anodic oxide for all tested conditions. Open-mouth nanotubes could be obtained over both phases under certain conditions, but their morphology strongly differed. Under optimized conditions, the nanotubes grown over the α-phase presented a well-defined, honeycomb-like, tubular structure with thin walls, while the ones grown over the β-phase were individual and much shorter with thicker walls and smaller inner diameters. The inner diameter of the TNTs grown over the α-phase increased in an almost linear way by increasing the applied potential, while the increase of the anodization time under a constant applied potential increased the inner diameter at a lower extension. The effect of the anodization potential and time was less marked on the TNTs grown over the β-phase.

The study of the early stages of the anodization process revealed that the initial compact oxide grew faster over the β-phase as the higher Al content of the α-phase caused its re-passivation. The higher solubility of the V-rich oxide led also to earlier pores formation over the β-phase. The trend was inverted once the pores formed over the compact oxide of the α-phase. The growth rate of the α-phase TNTs was higher than that of the β-phase ones, leading to the formation of longer, well defined nanotubes with thinner walls.

## Figures and Tables

**Table 1:** EDXS analyses results in %wt. (Figure 1a).

Element	Alloy	α	β
Ti	89.71	90.72	81.83
Al	6.42	6.47	4.79
V	3.87	2.56	13.38

**Table 2:** EDXS analyses results in %wt. performed on different sites on the surface of anodized specimens at 60 V for 120 min.

Element	Upper Surface	Cavity
O	44.6 ± 3.2	6.8 ± 5.1
Ti	49.3 ± 2.9	85.14 ± 3.8
Al	3.7 ± 0.2	2.1 ± 0.9
V	2.5 ± 0.2	6.0 ± 2.8

## Data Availability

The data that support the findings of this study are available from the corresponding author (M.L.) upon reasonable request.
